# Inhibition of NF-κB activation and MMP-9 secretion by plasma of human volunteers after ingestion of maritime pine bark extract (Pycnogenol)

**DOI:** 10.1186/1476-9255-3-1

**Published:** 2006-01-27

**Authors:** Tanja Grimm, Zuzana Chovanová, Jana Muchová, Katarína Sumegová, Anna Liptáková, Zdeňka Ďuračková, Petra Högger

**Affiliations:** 1 Institut für Pharmazie und Lebensmittelchemie, Bayerische Julius-Maximilians-Universität, Würzburg, Germany; 2 Department of Medical Chemistry, Biochemistry and Clinical Biochemistry, Faculty of Medicine, Comenius University, Bratislava, Slovak Republic

## Abstract

French maritime pine bark extract (Pycnogenol^®^) displays a variety of anti-inflammatory effects *in vivo*. Aim of this study was to determine whether human plasma after oral intake of Pycnogenol contains sufficient concentrations of active principles to inhibit key mediators of inflammation. Blood samples from seven healthy volunteers were obtained before and after five days administration of 200 mg Pycnogenol per day. Plasma samples statistically significantly inhibited matrix metalloproteinase 9 (MMP-9) release from human monocytes and NF-κB activation. Thus, we provide evidence that bioavailable active principles of Pycnogenol exert anti-inflammatory effects by inhibition of proinflammatory gene expression which is consistent with documented clinical observations. We suggest that our *ex vivo *method is suitable to substantiate molecular pharmacological mechanisms of complex plant extracts in a more focussed and rational way compared to *in vitro *studies by taking into account the processes of absorption and metabolism.

## Background

Pycnogenol is a standardized bark extract of the French maritime pine *Pinus pinaster *(Pycnogenol^®^, Horphag Research Ltd., UK). It comprises of a concentrate of pine bark constituents such as polyphenolic monomers, procyanidins and phenolic or cinnamic acids and their glycosides [1]. About 65–75 % of the Pycnogenol extract are procyanidins that consist of catechin and epicatechin subunits of varying chain lengths [1]. The quality of this extract is specified in the United States Pharmacopeia (USP 28) [2].

In human studies Pycnogenol revealed diverse anti-inflammatory actions [1]. Double-blind, placebo-controlled studies in asthma patients showed reduced plasma [3] or urine [4] leukotriene concentrations after Pycnogenol supplementation, while asthma symptom scores and pulmonary function improved. Symptoms of osteoarthritis as pain and immobility of joints decreased in a double-blind, placebo-controlled study [5]. Oral [6] and topical [7] application of Pycnogenol reduced inflammation and delayed skin-cancer formation following UV-radiation in controlled experiments in mice.

The anti-inflammatory mechanisms of maritime pine bark extract have been elucidated in a variety of *in vitro *and cell culture studies [8,9]. Additionally to its radical scavenging activity an inhibition of NF-κB-dependent gene expression and decrease of the activity of various pro-inflammatory mediators and adhesion molecules was observed after incubation of cells with the Pycnogenol extract [8,9]. This experimental *in vitro *design that pursues to uncover pharmacological effects by addition of plant extracts to cell cultures and subsequent measurement of cellular responses is widely employed. However, this methodology might inherit a couple of pitfalls.

Plant extracts often comprise of high molecular weight components that cannot be absorbed in the gastrointestinal tract and thus will never reach a target cell *in vivo*. Furthermore, there are examples of metabolites that are not present in the original extract, but are formed *in vivo *as a result of intestinal bacterial and/or hepatic metabolism. After ingestion of Pycnogenol, for example, two metabolites derived from catechin were detected in human urine, δ-(3,4-dihydroxy-phenyl)-γ-valerolactone and δ-(3-methoxy-4-hydroxy-phenyl)-γ-valerolactone [10]. Valerolactone derivatives were also found after ingestion of green tea [11]. These newly formed metabolites may display significant efficacy and contribute to the observed *in vivo *effects. We recently elucidated the cellular effects of δ-(3,4-dihydroxy-phenyl)-γ-valerolactone and δ-(3-methoxy-4-hydroxy-phenyl)-γ-valerolactone and uncovered an antioxidant activity as well as the potential to inhibit release and enzymatic activity of matrix metalloproteinase 9 (MMP-9) [12].

**Figure 1:**
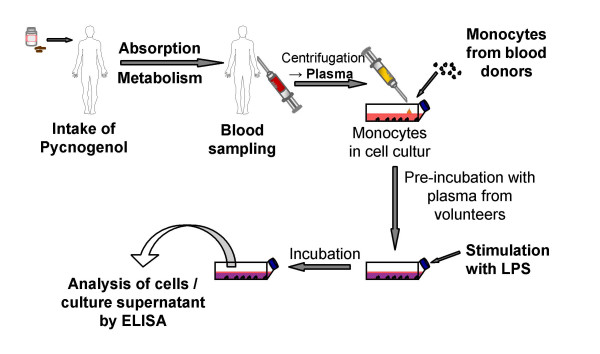
Schematic representation of the experimental procedure of the *ex vivo *experiments with human plasma incubated with monocytes.

Thus, pharmacokinetic issues of absorption and metabolism should be considered for valid identification of molecular pharmacological effects of plant extracts. A methodological approach that considers both the absorption and possible metabolism of plant extract components would involve laboratory animals or human volunteers who donate blood samples. These blood samples should contain all bioavailable active principles of the extract and allow an *ex vivo *analysis in all kind of molecular pharmacological effects in cell culture assays (Figure 1). There are only few examples of experimental settings described in literature that use this approach. Effects of nettle herb [13] or willow bark extract [14] on cytokine release and effect of *Harpagophytum *extract on eicosanoid biosynthesis [15] were elucidated in whole-blood assays of human volunteers after ingestion of the extract. Recently, a potent *ex vivo *anti-HIV activity was detected in sera of volunteers after administration of *Phyllanthus amarus *plant material [16].

The purpose of the present study was to determine molecular pharmacological effects of maritime pine bark extract *ex vivo *after intake of regular doses by human volunteers. Therefore, we obtained plasma samples before and after five days administration of Pycnogenol to seven healthy humans. These plasma samples were analyzed in two different experimental settings to evaluate the influence of bioavailable actives principles on cellular key components that contribute to inflammatory processes. We investigated a potential influence of the plasma samples on LPS-induced release of MMP-9 from human monocytes. Since MMP-9 induction and release might be initiated by NF-κB activation we also determined the effect of the plasma samples on LPS-induced NF-κB nuclear translocation.

## Methods

### Patients

Seven healthy volunteers (five female and two male) aged 18 to 30 years participated in this study. The study was approved by the ethical committee of the Comenius University's Faculty of Medicine, Bratislava, Slovak Republic, and all participants gave written informed consent. After 24 hours of a diet free of flavonoids (no vegetables, fruits and fruit juices or marmalades, tea, coffee, cocoa, wine and beer) blood samples were drawn to obtain basal values. Subsequently, the volunteers took tablets containing 200 mg standardized maritime pine bark extract (Pycnogenol^®^, Horphag Research Ltd., UK) every morning for five days to reach steady state conditions of constituents and/or metabolites of Pycnogenol. Four hours after the last intake of Pycnogenol on day five a second blood sample was obtained from each volunteer. Again, a 24 hour period of a diet free of flavonoids preceded this blood sampling. Blood samples were centrifuged and plasma was aliquoted, shock frozen and stored at -80°C until further analysis.

### Isolation and culture of human monocytes

Human monocytes were isolated from pooled blood cell suspensions (Bayerisches Rotes Kreuz, Wiesentheid, Germany) from different donors by Biocoll (Biochrom, Berlin, Germany) and subsequent Percoll (Pharmacia, Freiburg, Germany) density gradient centrifugation. Only blood cell suspensions of donors with blood type 0 were used for these experiments. The cells were first cultured overnight in Mc Coy's 5a modified medium (Biochrom, Berlin, Germany) supplemented with 15 % fetal calf plasma, 1 % penicilline/streptomycine, 1 % non-essential amino acids and 1 mM L-glutamine at a density of 5 × 10^6 ^cells/mL (NF-κB experiments) or 1 × 10^6 ^cells/mL (MMP-9 experiments) in a 6 % CO_2 _humidified atmosphere at 37°C (Hera cell incubator, Kendro Laboratory Products, Hanau, Germany). Cell experiments were performed in Multiwell™ 24-well cell culture plates, polystyrene, (BD Labware NJ, USA) with a final volume of 2.0 mL/well.

### Inhibition of MMP-9 release from human monocytes

Plasma samples obtained before and after Pycnogenol intake were diluted 1:1 with RPMI medium (Biochrom, Berlin, Germany; supplemented with 1 % penicilline/streptomycine, 1 % non-essential amino acids and 1 mM L-glutamine) and incubated with monocytes for one hour. Cells were then stimulated with 10 ng/ml LPS (Lipopolysaccharides (rough strains) from Salmonella minnesota Re 595, Sigma-Aldrich Inc., Taufkirchen, Germany) and incubated at 37°C for 48 hours. The number of viable cells was determined by counting living cells after staining with trypane blue. Plates were centrifuged (Megafuge 1.0 R, Kendro Laboratory Products) and cell culture supernatants were harvested, diluted 1:25 and assayed for total MMP-9 protein concentrations by ELISA (Quantikine™ assay, R&D Systems, Minneapolis, USA) according to manufacturer's protocol.

### Determination of NF-κB activation by ELISA

Plasma samples obtained before and after Pycnogenol intake were diluted 1:1 with RPMI medium as described above and incubated with monocytes overnight. Cells were then stimulated with 1 μg/mL LPS and incubated at 37°C for 60 minutes. After incubation the number of viable cells was determined by counting living cells after staining with trypane blue. Determination of free p65 in nuclear extracts was performed according to the manufacturer's protocol (ELISA-Kit NF-κB p65 ActivELISA™, Imgenex, CA, USA). The optical density of samples was determined using the microplate reader (Bio-Rad microplate reader, Benchmark CA, USA) set at 405 nm. Inhibitory effects of plasma constituents and metabolites after Pycnogenol intake were determined by comparing the p65 concentration of LPS-stimulated cells, incubated with plasma before and after Pycnogenol intake.

### Statistical analysis

Statistical analysis (Wilcoxon matched pairs signed rank test) was performed using the GraphPad prism software (GraphPad Software Inc., San Diego CA, USA). Significance was defined as p < 0.05.

## Results

Human monocytes were incubated with diluted plasma samples (dilution 1:1 with cell culture medium) obtained from seven healthy volunteers before and after ingestion of maritime pine bark extract (Figure 1). The viability of monocytes was not significantly influenced by plasma samples obtained from Pycnogenol treated subjects. In the MMP-9 experiments the number of viable monocytes was 2.49 ± 0.23 × 10^5 ^after incubation with samples obtained before Pycnogenol intake and 2.79 ± 0.26 × 10^5 ^after incubation with plasma obtained after 5 days Pycnogenol ingestion. Likewise, no difference was observed in the number of viable cells in the NF-κB experiments. The number of viable monocytes was 1.49 ± 0.29 × 10^6 ^and 1.70 ± 0.20 × 10^6 ^after incubation with samples obtained before and after 5 days Pycnogenol intake.

**Figure 2:**
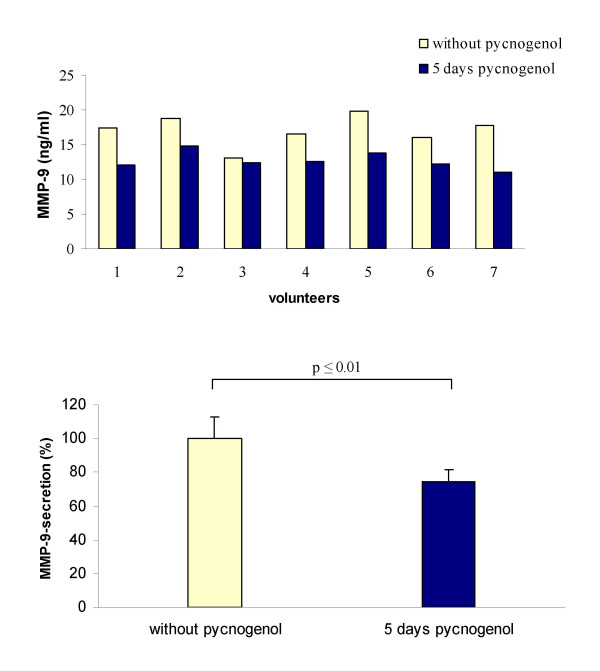
Inhibition of LPS-induced matrix metalloproteinase 9 (MMP-9) from human monocytes by plasma of seven volunteers before and after five days intake of 200 mg maritime pine bark extract (Pycnogenol). The upper panel shows concentrations of MMP-9 in cell culture supernatants of 2.5 × 10^5 ^viable cells after *ex vivo *incubation with the individual volunteers' plasma. The lower panel displays mean and standard deviation of percentage MMP-9 release. It statistically significantly reduced by plasma samples after administration of Pycnogenol (p < 0.01, Wilcoxon matched pairs signed rank test).

A statistically significant decrease of MMP-9 concentration in cell culture supernatant was induced by the plasma samples obtained after intake of Pycnogenol compared to basal values (Figure 2). The mean MMP-9 concentration after LPS challenge of monocytes incubated with volunteers' plasma samples obtained before Pycnogenol ingestion was 17.06 ± 2.17 ng/mL per 2.5 × 10^5 ^viable human monocytes. This concentration was reduced to 12.70 ± 1.24 ng/mL when monocytes were incubated with plasma obtained after 5 days Pycnogenol intake. This corresponded to a mean decrease in MMP-9 concentration of 25 %. The plasma of all study participants exhibited an inhibitory effect on LPS-induced MMP-9 secretion, but interindividual variations were obvious. The inhibitory effect ranged from 4.6 % to 39 % inhibition.

**Figure 3:**
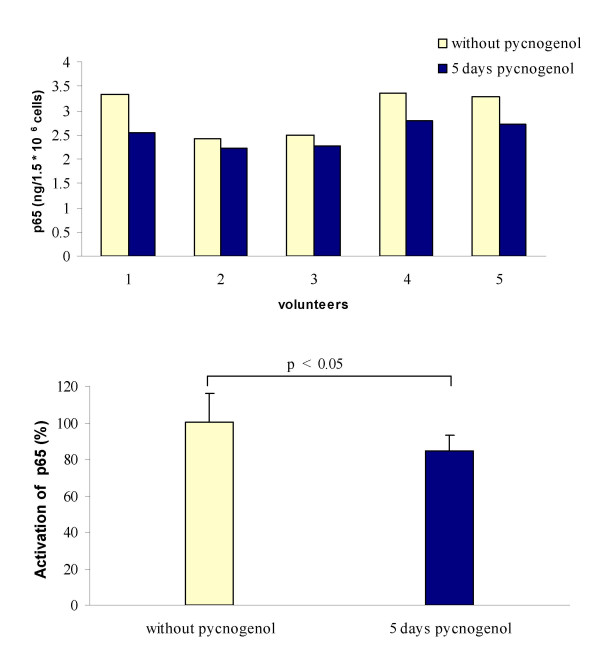
Inhibition of LPS-induced NF-κB activation by plasma of five volunteers before and after five days intake of 200 mg maritime pine bark extract (Pycnogenol). The upper panel shows concentrations of p65 was determined in nuclear extracts of 1.5 × 10^6 ^viable human monocytes after *ex vivo *incubation with the individual volunteers' plasma. The lower panel displays mean and standard deviation of percentage nuclear concentration of p65. It was statistically significantly reduced by plasma samples after administration of Pycnogenol (p < 0.05, Wilcoxon matched pairs signed rank test).

A statistically significant reduction of nuclear p65 concentration was observed when human monocytes were exposed to plasma (dilution 1:1 with cell culture medium) obtained after intake of Pycnogenol compared to basal values (Figure 3). For this experiment, sufficient volumes of plasma were only available from five volunteers; two plasma samples had been used up for repetition experiments after cell culture contamination. The mean nuclear p65 concentration after LPS challenge was 2.98 ± 0.48 ng per 1.5 × 10^6 ^viable human monocytes. This nuclear concentration was reduced to 2.51 ± 0.26 ng per 1.5 × 10^6 ^viable cells when monocytes were incubated with plasma obtained after 5 days Pycnogenol intake. The plasma of all study five participants exhibited an inhibitory effect on LPS-induced NF-κB activation, but interindividual variations were obvious. The inhibitory effect ranged from 6 % to 25 % with a mean of 15.5 % inhibition.

**Figure 4:**
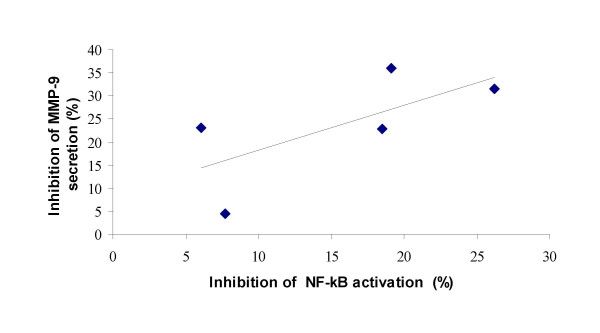
Correlation of inhibition of LPS-induced NF-κB activation and MMP-9 release by matching plasma samples of five volunteers before and after five days intake. Coefficient of correlation was 0.6 (Spearman rank correlation coefficient).

For five volunteers whose plasma samples were used for both the MMP-9 secretion and NF-κB experiments a correlation of their plasmas' inhibitory activity on MMP-9 secretion and NF-κB nuclear translocation was calculated (Figure 4). The correlation (Spearman rank correlation coefficient) was positive (r = 0.6) though not statistically significant due to limited number of samples.

## Discussion

Plant extracts may display a variety of interesting pharmacological effects *in vivo*. The bioefficacy of plant extracts is increasingly tested and documented in clinical intervention studies [17]. While the efficacy of extracts is observed with increasing interest the elucidation of the molecular basis of biological or clinical effects remains a challenge. Usually plant extracts comprise of a complex mixture of various components and often enough it is not clear whether a single compound or a mixture of related compounds is responsible for the effects.

The standardized maritime pine bark extract Pycnogenol has documented clinical anti-inflammatory activities [1,9]. In earlier studies we determined that the extract's metabolites δ-(3,4-dihydroxy-phenyl)-γ-valerolactone and δ-(3-methoxy-4-hydroxy-phenyl)-γ-valerolactone exhibited inhibitory activity on LPS-induced secretion of matrix metalloproteinase 9 (MMP-9) from human monocytes [12]. However, so far it remained elusive whether sufficiently high *in vivo *concentrations of any active extract components would be achieved after peroral intake of Pycnogenol. In the present study we applied an *ex vivo *methodology that takes absorption and metabolism of the extract into account. The plasma samples obtained from volunteers after ingestion of Pycnogenol were expected to contain active extract components that should attenuate inflammatory processes.

Indeed, we observed a statistically significant mean decrease of about 25 % in MMP-9 release when LPS-activated human monocytes were exposed to plasma of volunteers after repeated intake of Pycnogenol. The matrix degrading enzyme MMP-9 is highly expressed at sites of inflammation and contributes to the pathogenesis of various chronic inflammatory diseases. In asthma MMP-9 is upregulated and involved in remodeling processes [18-20]. MMP-9 also facilitates recruitment of inflammatory cells such as eosinophils and neutrophils across basement membranes [18]. Expression of MMP-9 was negatively correlated with pulmonary function in asthmatic patients [20]. As Pycnogenol has been reported to attenuate signs of inflammation in asthma patients [3,4] we now provide first evidence that this anti-inflammatory *in vivo *effect might be at least partially attributed to reduced MMP-9 secretion on a molecular level.

NF-κB is a molecule with a master function in inflammatory cytokine induction. It is also involved in regulation of immune functions, cell cycle control and apoptosis [21]. Upon nuclear translocation in response to an inflammatory stimulus it regulates various genes coding for proinflammatory mediators. We found that plasma of Pycnogenol treated volunteers statistically significantly inhibited NF-κB activation in LPS-stimulated monocytes by about 15 %. Though this effect is rather moderate it might well contribute to the anti-inflammatory effects of Pycnogenol in patients. Interestingly, NF-κB is also involved in MMP-9 expression [22,23]. Consistent with these reports we observed a positive correlation between inhibitory activity of matched plasma samples on MMP-9 and NF-κB. *In vitro *inhibition of NF-κB activation by plant extracts or constituents has been reported repeatedly [24]. Blocking IκB kinase activity has been reported as the underlying mechanism for restricting NF-κB activation by green tea polyphenols [25]. The mechanism of *ex vivo *inhibition of NF-κB nuclear translocation by plasma containing bioavailable active principles after Pycnogenol ingestion has yet to be identified.

To summarize, regular doses of perorally administered French maritime pine bark extract moderately inhibited NF-κB activation and MMP-9 secretion *ex vivo*. Since the plasma samples of the volunteers were diluted 1:1 with cell culture medium before incubation with the monocytes it can be assumed that *in vivo *effects might be even more pronounced. The observed *ex vivo *effects with plasma of volunteers after Pycnogenol intake are consistent with reported clinical anti-inflammatory effects *in vivo*. The next challenge will be to identify the responsible active principle(s) in the plasma samples. After all, however, the suggested methodology is a rational and focussed technique to explain biological effects from *in vivo *studies on a molecular pharmacological basis. The next target will be to link pharmacodynamic data with pharmacokinetics and to identify the active component(s) in plasma samples.

## Competing interests

This work was supported by a research grant of Horphag Research. Z.D. was additionally supported by a VEGA grant of the Ministry of Education of Slovak Republic and by Mind & Health civil association.

## Authors' contributions

T.G. carried out all experiments with the plasma samples and the data analysis.

Z.C. recruited the volunteers and organized the study, prepared the technical documentation for blood sampling.

J.M. and K.S. took care of the volunteers and performed blood sampling and processed samples according to the protocol.

A.L. prepared the project and processed blood samples.

Z.D. contributed to planning of the design and execution of the project and wrote the ethic's committee application.

P.H. conceived of and designed the study and wrote the manuscript.

All authors read and approved the final manuscript.
